# The Role of Mobile Apps in Obesity Management: Systematic Review and Meta-Analysis

**DOI:** 10.2196/66887

**Published:** 2025-05-06

**Authors:** Carmelo Pujia, Yvelise Ferro, Elisa Mazza, Samantha Maurotti, Tiziana Montalcini, Arturo Pujia

**Affiliations:** 1 OU Clinical Nutrition Renato Dulbecco Hospital Catanzaro Italy; 2 Department of Medical and Surgical Sciences University Magna Græcia Catanzaro Italy; 3 Department of Clinical and Experimental Medicine University Magna Græcia Catanzaro Italy; 4 Research Center for the Prevention and Treatment of Metabolic Diseases University Magna Græcia Catanzaro Italy

**Keywords:** smartphone apps, weight loss, weight management, body composition, obesity, meta-analysis, mHealth, mobile health, digital health, smartphone, telehealth, telemedicine, systematic review, public health, overweight, comorbidity, BMI

## Abstract

**Background:**

Obesity is a growing public health concern worldwide, significantly contributing to premature mortality and noncommunicable diseases. Weight reduction through lifestyle interventions, including diet and physical activity, is the primary approach to combating obesity, with studies showing that a 5% to 10% reduction in body weight can notably reduce obesity-related complications. Recently, smartphone apps have emerged as popular tools to aid in weight loss. However, the effectiveness of smartphone-only apps for weight management in people with overweight or obesity without comorbidities remains unclear.

**Objective:**

This meta-analysis aims to evaluate the efficacy of these apps in supporting weight loss and improving body composition in such populations.

**Methods:**

A systematic review and meta-analysis were conducted following PRISMA (Preferred Reporting Items for Systematic Reviews and Meta-Analyses) guidelines, with a search across databases including PubMed, Scopus, Cochrane Library, and others. The inclusion criteria were randomized controlled trials involving adults (aged ≥18 years) with overweight or obesity (BMI≥25 kg/m^2^) and assessing the use of smartphone-only apps for weight loss. Studies using additional devices or involving participants with comorbidities were excluded. Data extraction focused on weight loss, BMI, waist circumference, and body fat percentage, and the risk of bias was assessed using the Revised Cochrane Risk-of-Bias tool.

**Results:**

A total of 11 randomized controlled trials with 1717 participants were included in the meta-analysis. The interventions, lasting between 60 days and 12 months, involved diet and exercise monitoring via smartphone apps. At 4-6 months of follow-up, app-based interventions significantly reduced body weight (standardized mean difference –0.33, 95% CI –0.48 to –0.17; *P*<.001; *I*^2^=49%) and BMI (mean difference [MD] –0.76, 95% CI –1.42 to –0.10; *P*=.02). Reductions in body fat percentage were also observed at 3 months (MD –0.79, 95% CI –1.38 to –0.20; *P*=.009) and between 4 and 6 months (MD –0.46, 95% CI –0.71 to –0.20; *P*<.001). However, no significant effects on waist circumference were noted (*P*=.07).

**Conclusions:**

Smartphone apps demonstrate a modest but statistically significant effect on weight loss and BMI reduction over a 4- to 6-month period in individuals with overweight or obesity. The effectiveness of these interventions appears limited beyond 6 months, with a tendency for weight regain. Many apps lack the personalized support necessary to sustain long-term weight loss, contributing to high dropout rates. Future development of weight loss apps should focus on enhanced customization to improve user adherence and long-term outcomes.

**Trial Registration:**

PROSPERO CRD42024570999; https://tinyurl.com/2xw6j4fy

## Introduction

Obesity represents one of the major public health challenges of the 21st century. The World Health Organization [1] defines obesity as an abnormal or excessive accumulation of fat that may impair health. Globally, the prevalence of people with obesity is rising steadily, which is a big problem for health, economy, and society. In the United States, projections show that by 2050, the number of adults who are overweight or obese will increase from 172 million in 2021 to 213 million. This trend not only affects health care spending, with direct costs potentially reaching up to US $481 billion in 2016, but also leads to higher indirect costs due to lower productivity and work incapacity. The increase in obesity rates by 158.4% among male teens and 185.9% among female teens from 1990 to 2021 highlights the urgent need for effective preventive interventions [2]. This rise can be attributed to multiple factors, including lifestyle changes, unhealthy diets, physical inactivity, and socioeconomic determinants [3]. Obesity has a tremendous impact on public health, being responsible for millions of premature deaths each year. Moreover, it represents a significant risk factor for several noncommunicable diseases [4] such as type 2 diabetes, cardiovascular diseases, and cancers [2], which involve medical care, drugs, and hospital stays.

Tackling the obesity epidemic is thus a public health priority. Preventive interventions that promote healthy lifestyles are essential for reducing the incidence of obesity [5]. Promoting a balanced diet, combined with regular physical activity, is considered the most effective strategy for preventing and treating obesity. It is known that body weight, BMI, waist circumference (WC), and body fat percentage—all key indicators of obesity and general health status [6]—can differ significantly by age and between ethnicities [6-8]. Several studies have shown that a 5% to 10% reduction in body weight can lead to a significant decrease in obesity-related complications [9-13]. Recently, pharmacological treatment has gained popularity in addressing obesity. However, these medications are often associated with side effects and high costs, limiting access for part of the population [14]. The exponential growth of technology over the last decade offers promising tools for managing obesity through enhanced monitoring and personalized interventions [15,16]. Mobile health (mHealth) apps are playing an important role in preventive health care and helping to reduce the burden on health care organizations. The global mHealth apps market will grow from US $8 billion in 2018 to US $111.1 billion by 2025 [17]. In the United States, a national survey reported that 58.23% of the population uses mHealth apps [18]. However, challenges such as user engagement, data privacy, scientific validation, and integration into clinical practice highlight the need for balanced approaches and ongoing evaluation [15]. Since 2013, numerous clinical trials have measured the short- and long-term effects of weight loss apps. Systematic reviews and meta-analyses have examined studies up to 2022, including individuals with normal weight [19-21], patients with or without comorbidities (eg, cardiovascular diseases and metabolic syndromes) [22], as well as interventions combining smartphone apps with devices (eg, smartwatches, pedometers, or connected scales) or incorporating individualized nutritional counseling [23,24]. However, these inclusion criteria, with the recruitment of individuals with various characteristics, have made it unclear how effective the use of smartphone-only apps is for patients with overweight or obesity.

Therefore, we decided to exclude studies that included individuals with comorbidities other than obesity as well as those in which smartphone apps only provided reminders via SMS text messages or involved the use of additional devices (wearable or nonwearable). This choice was made to ensure greater homogeneity, minimize potential confounding factors related to comorbidities and additional tools, and isolate the specific effectiveness of smartphone-only apps in weight management.

Thus, the objective of this study was to evaluate the effects of nutritional interventions using exclusively smartphone apps on weight loss and body composition in patients with overweight or obesity without comorbidities and to update the current knowledge base. These findings may provide new insights to identify areas of improvement for smartphone apps to prevent and treat obesity and contribute to the development of innovative mobile apps to overcome the problems highlighted in the existing literature.

## Methods

### Selection and Search Strategy

This review was conducted following the guidelines of the PRISMA (Preferred Reporting Items for Systematic Reviews and Meta-Analyses) [25] (Multimedia Appendix 1). The protocol was registered in PROSPERO (CRD42024570999, accessed on August 2, 2024). Studies were identified through searches of the following electronic databases: CINAHL, Embase, PsycINFO, PubMed, Scopus, The Cochrane Library, and Web of Science. The search was conducted without time or language restrictions. The research question posed was “Is the use of smartphone apps effective for weight loss?” The studies were searched using specific terms related to the use of smartphone apps (“smartphone application,” “mobile application,” “app,” and “m-health”) and body weight management (“obesity,” “overweight,” “body weight,” “weight loss,” and “weight management”; Multimedia Appendix 2). To ensure the selection of relevant and high-quality studies, the search terms also included keywords related to randomized clinical trials (“randomized,” “randomised,” and “control group”; Multimedia Appendix 2). The last search was conducted from the inception of the electronic databases up until July 23, 2024.

### Inclusion and Exclusion Criteria

This review was designed to evaluate the effectiveness of smartphone-only interventions (apps) for weight loss. In particular, for the purposes of this study, we considered all weight loss apps, regardless of their specific features, such as goal planning, self-monitoring, motivational support, health education, and artificial intelligence (AI) integration. These key features of the apps included in the meta-analysis are detailed in Multimedia Appendix 3 [26-36].

The review included only studies that (1) involved adult participants (aged ≥18 years), (2) included individuals with overweight or obesity (BMI≥25 kg/m^2^), (3) evaluated the effectiveness of a smartphone app, and (4) reported weight loss as one of the outcomes. Only randomized controlled trials (RCTs) were included, along with studies that used either a nutritional intervention or no intervention (free diet) as a control.

Studies were excluded if they involved participants who did not have overweight or obesity or who had other comorbidities. Additionally, studies that did not follow an RCT design (eg, pilot studies or secondary analyses of RCTs, in which case the original RCT was retrieved), studies using smartphone apps providing only text message reminders via SMS, and studies where smartphone apps were used in combination with wearable devices (eg, step counters, smartwatches to manage or promote physical activity, or electronic weighing scales connected to the mobile app) were also excluded.

Furthermore, any studies not published in English were excluded. Systematic reviews and meta-analyses that assessed the use of smartphone apps for weight loss were examined to identify additional papers not captured in the initial database search. All extracted studies were reviewed, and the titles and abstracts were screened by 2 independent researchers (AP and EM) using the Rayyan application for systematic reviews to remove duplicates and assess eligibility based on the inclusion criteria. Any rejected papers were reviewed by a third researcher (SM) to confirm or refute exclusion. Disagreements between the 2 researchers were evaluated by the third researcher. Finally, the full texts of all papers were read independently by the same authors and further discussed.

### Data Extraction and Quality Assessment

Data extraction was independently performed by 2 researchers (CP and YF) using a Microsoft Excel spreadsheet template that collected information on the following criteria: authors; year of publication; country; sample size; sample characteristics; mean baseline BMI; mean population age; type of intervention and control; intervention duration; follow-up points; whether the study was funded; conflicts of interest; and study outcomes reported as either mean (SD), mean (SE), or mean difference (MD; 95% CI). The quality of the studies included in the meta-analysis was independently assessed using the Revised Cochrane Risk-of-Bias Tool for Randomized Trials. Based on the study methodology, the quality of the studies was rated as low, unclear, or high risk of bias. The following domains were assessed to determine study quality: randomization process, allocation concealment, blinding of participants and personnel to the interventions, blinding of outcome assessment, handling of missing data, and reporting of end-point outcomes in the study [37]. Two independent researchers (AP and EM) evaluated the risk of bias in the included studies using standardized data extraction tables. Any discrepancies were resolved through discussion with a third researcher (SM) until a consensus was reached.

### Statistical Analysis

The meta-analysis was conducted using all studies that were homogeneous in terms of interventions, reported outcomes, and included information on the end points analyzed in this study. Data reported as SE and CI were converted to SD. Additionally, if body weight was reported in pounds, it was converted to kilograms. The effect size on weight measured before 3 months was estimated using the weighted mean difference, while the standardized mean difference (SMD) was used for subsequent evaluations. For secondary outcomes such as BMI, WC, and body fat percentage, the effect size was estimated using weighted mean difference. All analyses were conducted using random-effects models. Heterogeneity between studies was estimated using the chi-square test and quantified using the *I*^2^ inconsistency measure, where 25%, 50%, and 75% indicate low, moderate, and high heterogeneity, respectively [38]. Values above 50% indicate substantial heterogeneity [39]. Finally, funnel plots were used as graphical tools to assess study precision and systematic heterogeneity. A symmetric funnel plot suggested that publication bias was less likely, while an asymmetric plot indicated publication bias. All statistical analyses were performed using Review Manager software (version 5.4; Cochrane Training).

## Results

**Figure 1:**
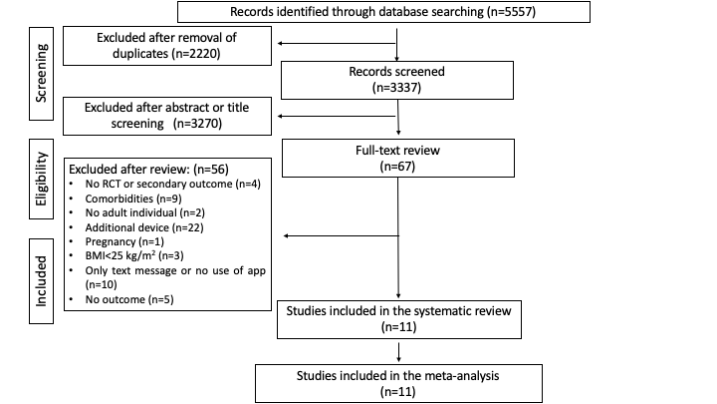
PRISMA (Preferred Reporting Items for Systematic Reviews and Meta-Analyses) flowchart of the research strategy. RCT: randomized controlled trial.

A total of 5557 papers were identified for this study. After removing 2220 duplicate papers, 3337 papers were screened for inclusion based on their title and abstract (Figure 1). After 3270 papers were excluded, the 67 papers that remained underwent full-text assessment. A total of 56 papers were excluded (Figure 1), with 11 studies included in the meta-analysis [26-36].

The main characteristics of the included studies, the types of interventions, as well as their effects on body weight and body composition are reported in Table 1.

A total of 11 studies published between 2013 and 2023 were included. Among the evaluated studies, 6 were conducted in Europe [26-28,30,33,35], 2 in the United States [31,36], 2 in Asia [29,34], and 1 in Turkey [32]. Most studies represented both genders, with a higher enrollment of female participants. The 11 studies included a total of 1717 patients with overweight and obesity. Sample sizes varied from 20 to 566 participants. The average age of participants ranged from 25 to 47 years, with a mean BMI between 27.9 and 36.3 kg/m^2^. Only one study [27] reported the average weight of the population without indicating the BMI. Dropout rates varied from 5% to 62%, with only 3 studies reporting no dropouts [29,32,35].

The interventions were primarily app-based, including real-time diet and exercise monitoring, personalized messages, immediate feedback, and lifestyle improvement advice. In 4 studies, the control group received no treatment [27,29,30,33]; in 3 studies, the control group followed standard nutritional treatment [26,31,32]; and in 2 studies, a paper food diary was used to record calorie intake [28,34]. In only 1 study was the intervention group compared to a wearable “Bite Counter” device [36]. The duration of the interventions ranged from 60 days to 12 months, with follow-ups from 4 weeks to 12 months.

In 4 studies, no significant differences in weight reduction emerged [26,29,32,34], while other studies, such as Balk-Møller et al [27], reported significant reductions in body weight and WC in the intervention group. Studies like Carter et al [28] highlighted significant reductions in weight, BMI, and fat mass. Similar results were reported in other studies, such as Gemesi et al [30], which showed maintained weight reduction over time. Overall, most interventions demonstrated a positive effect on weight loss and improvements in other health parameters, although results varied based on the type of intervention and study duration.

**Figure 2:**
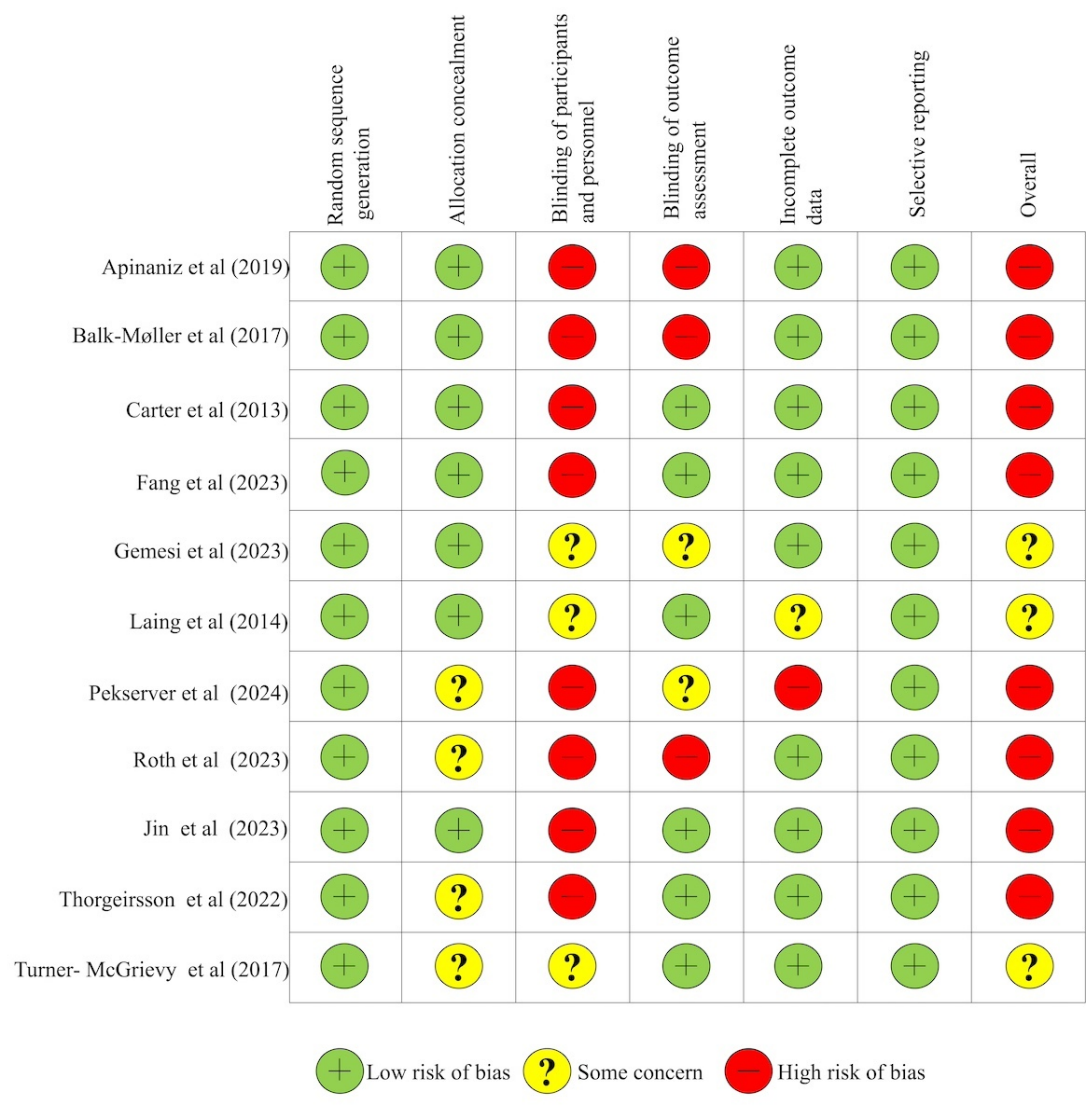
Assessment of the methodological quality of included studies.

Figure 2 [26-36] presents the assessment of bias risk for individual studies. Most studies (10/11, 91%) showed an acceptable randomization process. However, most papers (8/11, 73%) were assessed with a high risk of performance bias due to difficulties in implementing double-blinding, which is common in this type of research. Additionally, in some studies, the assessment of body weight (the primary outcome) relied on self-reporting.

Due to differences in follow-up durations across the studies, the results were analyzed at <3, 3, 4-6, and 9-12 months, where possible. All 11 included papers reported outcomes on weight loss. Specifically, only 2 studies, involving a total of 126 patients, assessed changes in body weight before 3 months [26,29]; 6 studies, totaling 704 patients, evaluated changes in body weight at 3 months [26,30-33,36]; 8 studies, with a total of 1466 patients, assessed changes in body weight between 4 and 6 months [26-28,30,31,33,35,36]; and finally, 2 studies, comprising a total of 664 patients, evaluated changes in body weight between 9 and 12 months [27,33] (Table 2).

**Table 1:** Study characteristics.

Study	Participants	Age (years), mean (SD)	Device	Duration	Intervention	Comparator	Objectives	Results
Apiñaniz et al (2019) [26] (Spain)	110 (54 intervention, 56 control)	38.5 (5)	Smartphone app AKTIDIET	6 months	Nutritional guidance reinforcement, aerobic exercise program, dietary intake logging, video explanations of exercises, motivational text messages	Standard nutritional treatment	Weight reduction and changes in cholesterol, HbA_1c_^a^, and systolic blood pressure	No significant changes in weight, cholesterol, or systolic blood pressure; significant reduction in HbA_1c_ favoring control
Balk-Møller et al (2017) [27] (Denmark)	566 (355 intervention, 211 control)	47 (10)	Web and smartphone app SoSu-life	38 weeks	Daily diet and exercise logging with personalized feedback	No intervention	Weight reduction; changes in waist circumference, fat mass, cholesterol, blood pressure, and behavior change	Greater decrease in body weight and fat percentage in the SoSu-life group
Carter et al (2013) [28] (United Kingdom)	128 (43 smartphone, 42 website, 43 control)	42.2 (9)	Website and smartphone app My Meal Mate	Baseline, 6 weeks, 6 months	Self-monitoring of diet and physical activity with weekly personalized messages	Paper food diary	Feasibility and acceptability of adherence to the study; changes in anthropometric parameters	Significant weight reduction in the smartphone group
Fang et al (2023) [29] (Taiwan)	20 (10 intervention, 10 control)	42.0 (8.5)	Smartphone app CogniNU	60 days	Food image recognition algorithm for identifying ingredients and estimating nutritional values	No intervention	Weight reduction, impact on eating behavior, and mood	No significant weight reduction; improvements in mindful eating behavior
Gemesi et al (2024) [30] (Germany)	168 (84 intervention, 84 control)	46.8 (11)	Smartphone app *DiHA*-*Oviva Direkt für Adipositas*	6 months	Weekly educational content on lifestyle for weight loss	No intervention	Weight reduction and quality of life	Significant weight reduction in the intervention group
Laing et al (2014) [31] (United States)	212 (105 intervention, 107 control)	43.1 (14.2)	Smartphone app MyFitnessPal	6 months	Self-monitoring of calories and physical activity	Standard nutritional treatment	Weight reduction and changes in blood pressure	Weight loss in the intervention group; weight gain in the control group
Peksever et al (2024) [32] (Turkey)	79 (39 intervention, 40 control)	34.7 (14)	Smartphone app MOtiVE	3 months	Personalized text, visual, and video messages for diet management	Standard nutritional treatment	Weight loss, quality of life, and eating behavior	No significant difference in weight loss between groups
Roth et al (2023) [33] (Finland)	150 (77 intervention, 73 control)	43.4 (10.9)	Smartphone app Zanadio	12 months	Multimodal approach to support weight loss	No intervention	Weight reduction, changes in fat mass, quality of life, and well-being	Significant weight loss in the intervention group
Jin et al (2023) [34] (Korea)	57 (30 intervention, 27 control)	25.4 (4.9)	Smartphone app Noom Coach	12 weeks	Daily calorie intake self-monitoring	Food diary	Reduction of anthropometric and metabolic parameters	No significant difference in weight reduction
Thorgeirsson et al (2022) [35] (Iceland)	146 (95 intervention, 51 control)	46.8 (11.7)	Smartphone app Sidekick	4 months	Promoting healthy behaviors through goal-setting and self-monitoring	Standard physical activity program	Weight reduction	Significant weight loss in the intervention group
Turner-McGrievy et al (2017) [36] (United States)	81 (42 intervention, 39 control)	48 (12)	Smartphone app FatSecret	6 months	Daily calorie intake monitoring	Bite Counter wearable device	Weight reduction	Greater weight loss in the app group compared to the Bite group

^a^HbA_1c_: hemoglobin A_1c_.

**Figure 3:**
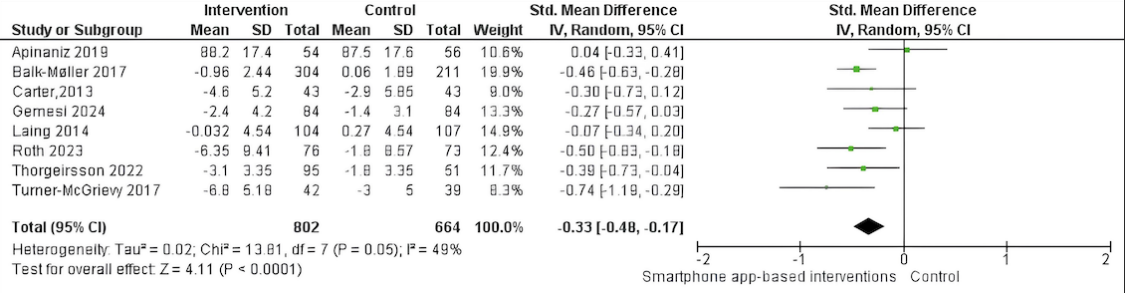
Forest plots of the effects of smartphone app–based interventions with a duration between 4 and 6 months on body weight.

As shown in Table 2 and Figure 3 [26-28,30,31,33,35,36], smartphone app–based interventions with a duration between 4 and 6 months demonstrated a statistically significant effect on body weight reduction in individuals with overweight or obesity (n=8 studies; n=1466 participants; SMD −0.33, 95% CI –0.48 to –0.17; *P*<.001; *I*^2^=49%).

**Table 2:** Summary of meta-analysis results for each outcome at different time points.

Follow-up			Participants, n		MD^a^ (95% CI)		*P* value	*I*^2^ (%)		Clinical relevance^b^
**Body weight (kg)**										
	<3 months	126		–0.53 (–1.12 to 0.07)		.08		0	No	
	3 months	704		–0.25^c^ (–0.59 to 0.08)		.13		77	No	
	4-6 months	1466		–0.33^c^ (–0.48 to –0.17)		<.001		49	No	
	9-12 months	664		–0.52^c^ (–1.27 to 0.23)		.17		93	Yes	
**BMI (kg/m^2^)**										
	<3 months	126		–0.08 (–1.59 to 1.43)		.91		33	No	
	3 months	95		0.93 (–0.43 to 2.29)		.18		0	No	
	4-6 months	232		–0.76 (–1.42 to –0.10)		.02		59	Yes	
	9-12 months	―^d^		―		―		―	―	
**Waist circumference (cm)**										
	<3 months	―		―		―		―	―	
	3-6 months	552		–0.64 (–1.33 to 0.05)		.07		0	No	
	9-12 months	―		―		―		―	―	
**Adipose mass (%)**										
	<3 months	126		–0.58 (–1.62 to 0.46)		.28		0	N/A^e^	
	3 months	234		–0.79 (–1.38 to –0.20)		.009		0	N/A	
	4-6 months	701		–0.46 (–0.71 to –0.20)		<.001		0	N/A	
	9-12 months	―		―		―		―	―	

^a^MD: mean difference.

^b^Body weight reduction ≥5% (6%-10%); BMI reduction between 0.20 and 0.25 kg/m^2^ [40]; and waist circumference reduction between 3 and 6.8 cm [41].

^c^Standardized mean difference.

^d^Not available.

^e^N/A: not applicable.

**Figure 4:**
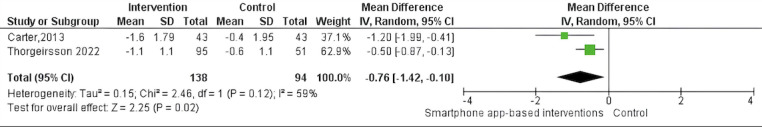
Forest plots of the effects of smartphone app–based interventions with a duration between 4 and 6 months on BMI.

No statistically significant reduction in body weight is reported before 3 months, at 3 months, and between 9 and 12 months of follow-up (all *P*<.05; Table 2). Regarding the effect on BMI, as shown in Table 2 and Figure 4 [28,35], smartphone app–based interventions with a duration between 4 and 6 months demonstrated a statistically significant effect on BMI reduction in individuals with overweight or obesity (n=2 studies; n=232 participants; MD –0.76, 95% CI –1.42 to –0.10; *P*=.02; *I*^2^=59%). No statistically significant reduction in BMI is reported before 3 months and at 3 months of follow-up (all *P*<.05; Table 2).

The effect on WC was evaluated in only 2 studies. As shown in Table 2, smartphone app–based interventions with a duration between 3 and 6 months did not demonstrate any statistically significant effect on WC reduction in individuals with overweight or obesity (n=2 studies; n=552 participants; MD –0.64, 95% CI –1.33 to 0.05; *P*=.07; *I*^2^=0%).

**Figure 5:**
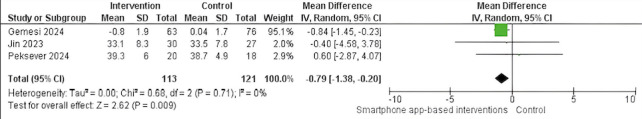
Forest plots of the effects of smartphone app–based interventions at 3 months on body fat percentage.

Finally, the effect of nutritional interventions using smartphone apps on the change in body fat percentage was assessed. No statistically significant reduction in body fat percentage was observed in individuals with overweight or obesity who had follow-up before 3 months (*P*=.28; Table 2). However, the use of the app resulted in a statistically significant reduction in body fat percentage at 3 months (n=3 studies; n=234 participants; MD –0.79, 95% CI –1.38 to –0.20; *P*=.009; *I*^2^=0%; Figure 5 [30,32,34]).

**Figure 6:**
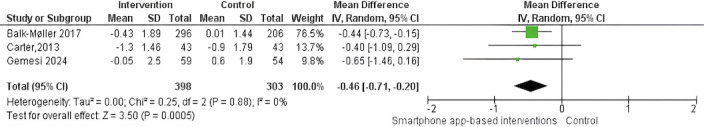
Forest plots of the effects of smartphone app–based interventions with a duration between 4 and 6 months on body fat percentage.

**Figure 7:**
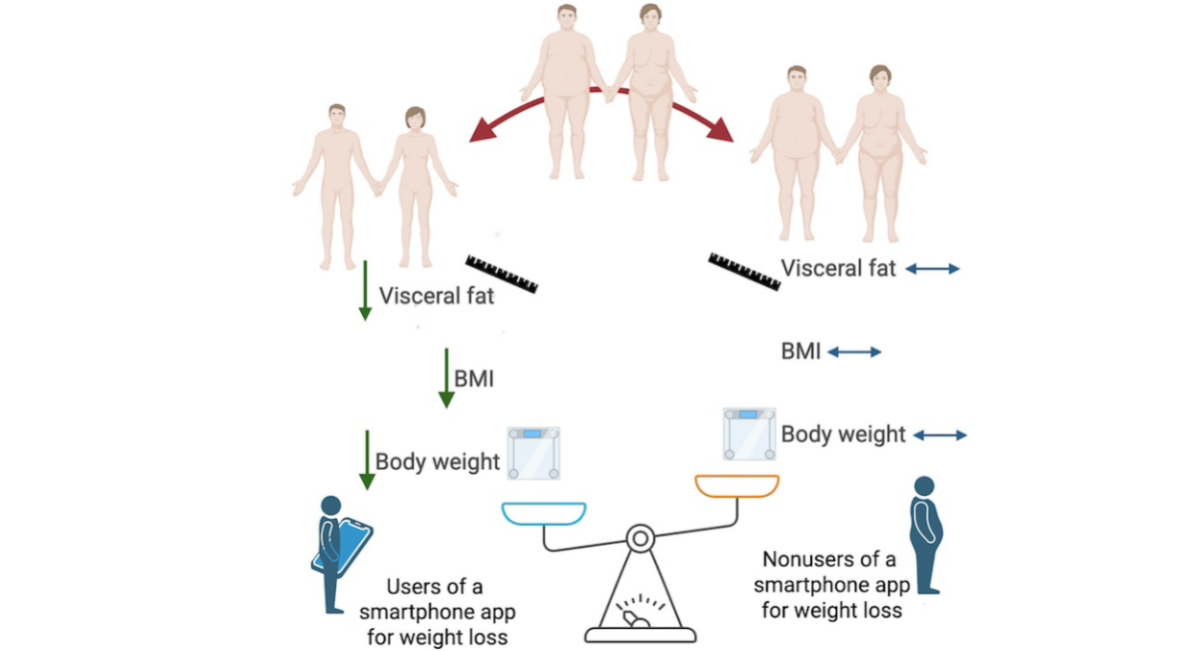
Effects of smartphone apps on weight and body composition.

A statistically significant reduction in body fat percentage is also confirmed between 4 and 6 months of follow-up after the use of smartphone apps (n=3 studies; n=701 participants; MD –0.46, 95% CI –0.71 to -0.20; *P*<.001; *I*^2^=0%; Figure 6 [27,28,30]). Finally, Figure 7 shows the main results obtained.

**Figure 8:**
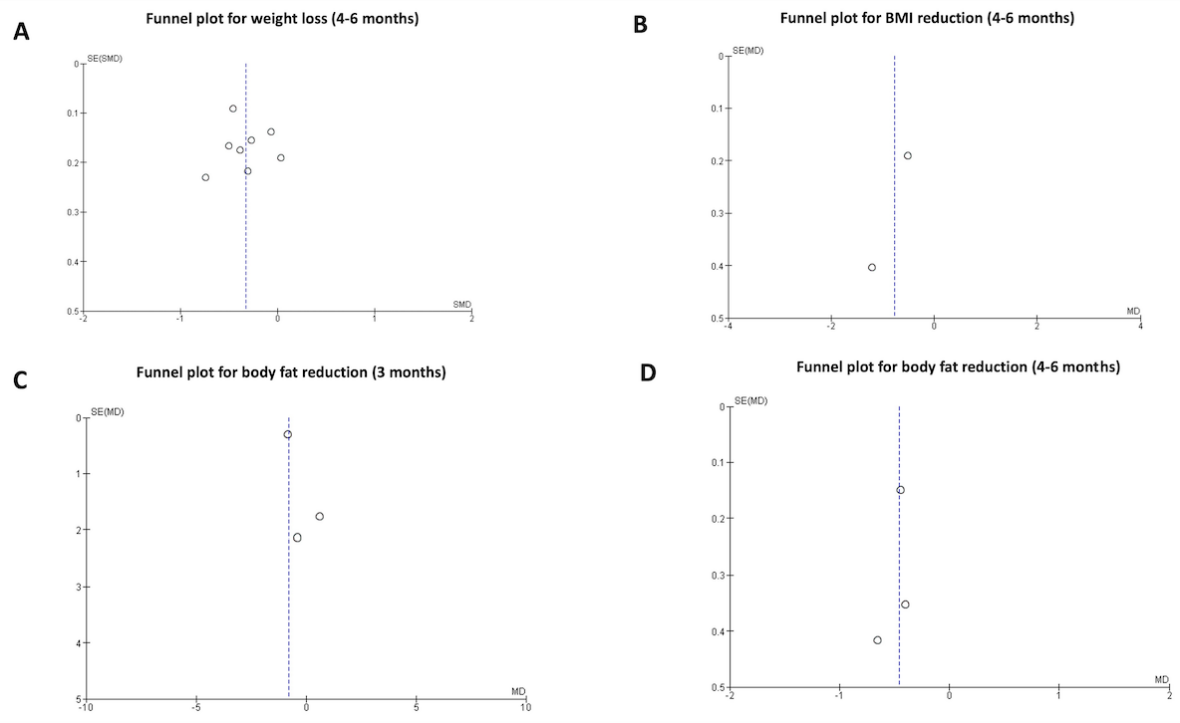
Funnel plots of the effects of smartphone app–based interventions on (A) body weight, (B) BMI, (C) body fat percentage at 3 months, and (D) between 4 and 6 months. MD: mean difference.

Figure 8 presents the funnel plots showing the distribution of studies from the main analyses of the meta-analysis. Funnel plots were used to identify potential publication bias and the symmetry of studies around the mean effect line. Visual analysis of the funnel plots suggests general symmetry among the studies.

## Discussion

### Principal Findings

This meta-analysis on studies evaluating the effects of weight loss apps stems from the need to explore an area of growing interest for both the scientific community and the general public. In an era where mobile technology is playing a central role in managing health and personal well-being—especially with the widespread use of smartphones and internet connectivity—the interest in these apps is motivated by their potential effectiveness in supporting individuals with weight management. These apps integrate behavioral strategies, monitoring, and nutritional education directly on mobile devices, making them accessible and customizable, particularly given the significant nonadherence of a large part of the population to international healthy lifestyle recommendations. This meta-analysis aims to deepen our understanding not only of the effectiveness of such tools but also to identify areas for improvement and current challenges, with the prospect of developing an innovative app that can address some of the issues found in the existing literature.

Most of the studies included in this meta-analysis reported results on weight loss at 3 and 6 months, but few explored the impact on other parameters such as BMI, WC, and body fat percentage. The results of the meta-analysis indicate that smartphone app–based interventions lasting between 4 and 6 months resulted in a significant reduction in body weight (n=8 studies; n=1466 participants; SMD −0.33, 95% CI –0.48 to –0.17; *P*<.001; *I*^2^=49%). However, no statistically significant reductions in body weight were observed before 3 months or in other analyzed time intervals. The weight loss observed in this meta-analysis is lower compared to 2 previous studies. One study reported significant weight losses of –1.99 and –2.8 kg at 3 and 6 months, respectively, analyzing a population consisting of both normal-weight individuals and those who were individuals with overweight or obesity [42]. Another study reported a weight reduction of –2.18 kg at 3 months and –1.63 kg at 12 months, but it included smartphone apps with additional components such as pedometers and weight scales as well as patients with overweight or obesity and metabolic conditions [43]. This meta-analysis specifically aimed to investigate the effect of exclusive use of smartphone apps on body weight in individuals with uncomplicated overweight and obesity, comparing it with standard nutritional treatments or no treatment at all. Regarding other outcomes, it was found that smartphone app interventions lasting between 4 and 6 months led to a significant reduction in BMI (–0.76 kg/m^2^). Additionally, there was a significant reduction in body fat percentage at 3 months (–0.79%) and between 4 and 6 months of intervention (–0.46%). This meta-analysis confirms that smartphone app–based interventions appear to be ineffective in promoting clinically significant weight loss, with minimal and unsustainable effects over the long term, consistent with existing literature. Indeed, many studies, including those by Unick et al [44] and Al Naabi et al [45], have highlighted that while weight loss apps are often effective in the short term, the ability to maintain achieved results diminishes over time. This phenomenon is primarily attributed to a lack of continuous personalized support and strategies to address stalls or regressions in the weight loss journey. Supporting this is the finding that approximately 75% of users discontinue use of these apps within a short period [46].

Several reasons for this trend have been analyzed in a review [47], with a key issue being the limited personalization of apps. Many apps rely on standardized approaches that do not adequately consider individual needs, such as pre-existing medical conditions, dietary preferences, or physical limitations. The lack of individualization can reduce the overall effectiveness of the app and increase the risk of dropout. This is supported by the analysis of studies in this meta-analysis, which indicates that most of the tested apps lack personalization, while better results are achieved by apps that incorporate some form of customization, such as the study by Carter et al [28], which provided personalized feedback and resulted in a nearly 5-kg reduction in weight at 6 months. Furthermore, many apps tend to focus on quantitative monitoring of weight and calories [48], neglecting psychological and emotional aspects, such as motivation and stress management, which influence long-term results. Indeed, it has been demonstrated that a lack of motivation can limit the acceptance and effectiveness of even the most well-designed mHealth apps [49].

Another key aspect in weight loss through the use of smartphone apps can be attributed to the level of app engagement. A recent study has indeed evaluated the effectiveness of engaging with mobile apps in achieving weight loss in adults with overweight and obesity in a real-world context [50]. In particular, after 12 weeks, as many as 53% of participants classified as active users (active days during the observation period is 100%) experienced a clinically significant weight loss (≥5%) compared to 20% of inactive users (in-app activity <33%) [50]. The same authors also demonstrated a dose-response effect, whereby an increase in in-app activity was associated with greater weight loss [50].

In addition, several studies have demonstrated that gamification makes the app more enjoyable and motivating to use [51], provides emotional support that sustains user motivation [51], and most importantly, helps users promote health improvements and achieve their goals such as weight loss and physical activity [52].

Furthermore, the significance of the app’s design should not be underestimated. It should appeal to users, meet their needs, and feature a straightforward interface that integrates seamlessly with other devices. Ideally, it should be free or without hidden costs [49].

Another important aspect for weight management apps is that they provide food recommendations that consider not only the nutritional needs of the users but also the food culture [53]. This involves ensuring that the foods suggested are not only healthy and appropriate for weight management but also accessible and compatible with local food preferences. For example, an app aimed at the Italian market could emphasize the use of foods typical of the Mediterranean diet, while in Asia, it could focus on low-fat diet options rich in rice and vegetables. In fact, many smartphone apps have too limited choice of pre-entered meals in their databases, which often do not include ethnic foods [54]. Furthermore, they should integrate algorithms that consider variations in body composition, such as the ratio of fat to lean mass, which can differ significantly between ethnicities [8]. These aspects are essential to ensure that the apps are used and effective for a wide range of users even in different contexts. Therefore, all strategies aimed at encouraging greater use of the app, and consequently success in weight loss, should be considered in order to develop more effective mobile apps for obesity management.

Finally, several studies have highlighted the lack of scientific validation of many of the apps currently on the market for weight loss [42]. This could potentially increase stress and anxiety, as well as promote poor body image, and maladaptive eating and exercise behaviors, especially in younger individuals [49,55]. A study on nutrition apps found that some nutritional values varied by up to 50% from those reported by the German Food Database [56]. Therefore, apps without scientific validation as well as those designed by nonmedical institutions often lack expert knowledge and unreliable and nontransparent content, raising concerns about the accuracy of health information and its use in health management [56,57]. This represents a problem both for users and health professionals who could recommend their use.

The identified challenges in the literature also represent opportunities for developing a new app that can address these limitations and incorporate features that users have identified as critical for acceptability, engagement, and use. In addition, new smartphone apps could be integrated with AI systems, which may enhance personalization by analyzing large datasets, including genetics, biomarkers, diet, and lifestyle, to create tailored nutritional and physical activity plans based on the user’s unique characteristics [56,58]. Leveraging AI algorithms, these apps may automatically adjust goals based on user progress and provide suggestions to enhance body weight management [59].

Furthermore, apps that incorporate the combined use of AI and behavioral science techniques (eg, rewards, challenges, positive reinforcement, automatic monitoring, and personalized reminders) can enhance adherence and motivation, thereby reducing the risk of abandonment of weight management apps [60-62]. AI algorithms can detect inconsistencies and errors in user-recorded data, thereby enhancing the accuracy of analyses and rendering the recommendations more effective [63].

Ultimately, AI can equip health care providers with predictive models to identify individuals who may have difficulty losing weight and identify patients at risk of obesity-related complications. This enables proactive interventions through personalized strategies, fostering precision medicine [63]. Thus, these new mobile apps for weight management could be integrated into primary care systems by, for example, providing patients with self-monitoring tools and personalized feedback [64], facilitating communication between doctor and patient, and reducing the workload of health care providers.

In this context, several studies have demonstrated that smartphone apps can be effective tools for both weight management and the management of chronic conditions such as diabetes and cardiovascular diseases [65,66]. Additionally, these approaches could be integrated into public and community health programs, enhancing obesity prevention through digital education strategies and continuous monitoring of dietary habits and physical activity. Indeed, several studies have demonstrated that mobile apps can aid in preventing obesity by promoting adherence to healthier eating patterns and increasing physical activity among children and adolescents [67].

In fact, it is widely recognized that younger populations are heavy users of technology, especially smartphones and apps [18,68]. For this reason, weight management apps are designed also with them in mind, with easy-to-use interfaces and interactive features that fit well with their daily technological habits and preferences.

New weight management apps that incorporate AI, among other features, must overcome several challenges, including data privacy protection, the need for human oversight, and the risk of excessive dependence on technology. Therefore, integrating AI with the support of health care professionals may be the key to maximizing benefits without compromising the human aspect of interventions.

### Recommendations

The results of this study highlight the limited effectiveness of smartphone apps in their current form and provide numerous insights for future research. In particular, future smartphone apps could provide long-term support through personalized assistance during challenging times, integrating digital coaching, web-based communities, notifications, and gamification to ensure dynamic and digital communication with users. Additionally, they could provide a highly personalized approach by adapting to the user’s dietary preferences, goals, and physical and psychological characteristics, using AI to deliver dynamic recommendations. They could integrate psychological and motivational support, such as mindfulness practices and emotional coaching, to address the emotional aspects of weight management. Furthermore, it is imperative that new apps for body weight management incorporate considerations for data management and privacy, the requirement for human supervision, and the risk of developing an overreliance on technology. Support from health care professionals may thus be crucial in optimizing the benefits of these apps while preserving the human element of the interventions. Finally, it is essential to ensure the scientific validation of these nutritional interventions by developing apps in collaboration with experts and conducting clinical testing to confirm their effectiveness and reliability.

### Strengths and Limitations

This study has several strengths. First, each step in the process of conducting this systematic review (ie, study selection, data extraction, and methodological assessment) was performed independently by different reviewers. Furthermore, the results obtained were reported as MD, with the exception of weight loss, which was expressed as an SMD, in order to provide useful and clinically relevant insights into the potential effects of smartphone apps on weight loss and changes in body composition. To warrant the rigor of this study, it was decided to evaluate and include only peer-reviewed papers, thereby excluding those found in the gray literature. This approach ensured greater robustness of the results, although it may have excluded some potentially significant papers. Finally, only studies using smartphone apps were included, while those using additional devices that could have influenced the results were excluded.

Furthermore, the analysis focused on the effects of the apps on individuals with overweight or obesity, excluding individuals with comorbidities to minimize bias in the outcomes of the meta-analysis. However, it is important to note that this study has several limitations. The number of studies included in the meta-analysis is limited, and the majority of them were assessed as having unclear or high risk of bias, primarily due to the challenges in blinding participants to the intervention. Furthermore, we excluded studies with additional tools; although this approach improves the methodological rigor and specificity of findings, it may limit generalizability to real-world and practice settings, where smartphone apps are often used with wearable devices. Similarly, the choice to exclude individuals with comorbidities may also reduce the generalizability of the findings, as obesity is often associated with metabolic and cardiovascular diseases. Moreover, this meta-analysis did not take into account other variables that could affect responses to weight loss, such as genetic, psychological, economic, and social factors, as well as gender differences [69]. Finally, the results of this meta-analysis may not be directly applicable to all global populations. The included RCTs were conducted primarily in Europe, the United States, and Asia, contexts characterized by differences in dietary patterns, lifestyle habits, and health care systems. Furthermore, the effectiveness of apps may vary based on access to technology and levels of digital literacy.

For these reasons, the results obtained in this study should be interpreted with caution; however, they provide numerous insights for future clinical research aimed at developing more effective smartphone apps for body weight management, including in more complex clinical settings, also considering their applicability to different populations and health systems.

### Conclusions

The current landscape of weight loss apps offers interesting insights but also presents significant challenges. Through this meta-analysis, the authors aim to provide a solid and critical knowledge base that can be used for developing new, more effective, and sustainable solutions over time. The lack of personalization and continuous support represents a significant shortcoming, indicating the need to develop more advanced apps tailored to individual needs. To enhance the effectiveness of these tools, future research should explore the integration of advanced features such as adaptive feedback driven by AI, which could analyze personal user data and provide dynamic recommendations for diet, physical activity, and motivational support. Additionally, gamification systems and digital notifications should be developed to increase engagement and persistence in app use. Beyond weight loss, future studies should assess additional parameters to determine app effectiveness, such as long-term adherence, changes in body composition, quality of life, relationship with food, and stress management. Furthermore, ensuring the scientific validation of these apps will be crucial, involving experts in nutrition and health technology to develop reliable, evidence-based tools. These improvements could not only enhance the impact of apps on weight management but also transform them into effective tools for health prevention and promotion, integrating into primary care pathways and public health strategies. In conclusion, by leveraging existing strengths and addressing the identified challenges, it is possible to design an app that more comprehensively meets user needs, providing personalized, scientifically validated support that can enhance long-term well-being.

## Appendix

Multimedia Appendix 1 PRISMA (Preferred Reporting Items for Systematic Reviews and Meta-Analyses) checklist.

Multimedia Appendix 2 Search strategy.

Multimedia Appendix 3 Key features of the apps included in the meta-analysis.

## Abbreviations

AI: artificial intelligence
MD: mean difference
mHealth: mobile health
RCT: randomized controlled trial
SMD: standardized mean difference
WC: waist circumference

## Data Availability

The datasets generated or analyzed during this study are available from the corresponding author on reasonable request.

## References

[1] Obesity and overweight. World Health Organization.

[2] GBD 2021 US Obesity Forecasting Collaborators (2024). National-level and state-level prevalence of overweight and obesity among children, adolescents, and adults in the USA, 1990-2021, and forecasts up to 2050. Lancet.

[3] Swinburn BA, Sacks G, Hall KD, McPherson K, Finegood DT, Moodie ML, Gortmaker SL (2011). The global obesity pandemic: shaped by global drivers and local environments. Lancet.

[4] Kloock S, Ziegler CG, Dischinger U (2023). Obesity and its comorbidities, current treatment options and future perspectives: challenging bariatric surgery?. Pharmacol Ther.

[5] Kumanyika SK, Obarzanek E, Stettler N, Bell R, Field AE, Fortmann SP, Franklin BA, Gillman MW, Lewis CE, Poston WC, Stevens J, Hong Y (2008). Population-based prevention of obesity: the need for comprehensive promotion of healthful eating, physical activity, and energy balance: a scientific statement from American Heart Association Council on Epidemiology and Prevention, Interdisciplinary Committee for Prevention (formerly the expert panel on population and prevention science). Circulation.

[6] Holmes CJ, Racette SB (2021). The utility of body composition assessment in nutrition and clinical practice: an overview of current methodology. Nutrients.

[7] De Lorenzo A, Itani L, El Ghoch M, Gualtieri P, Frank G, Raffaelli G, Pellegrini M, Di Renzo L (2024). Difference in body composition patterns between age groups in Italian individuals with overweight and obesity: when BMI becomes a misleading tool in nutritional settings. Nutrients.

[8] Lear SA, Kohli S, Bondy GP, Tchernof A, Sniderman AD (2009). Ethnic variation in fat and lean body mass and the association with insulin resistance. J Clin Endocrinol Metab.

[9] Knowler WC, Barrett-Connor E, Fowler SE, Hamman RF, Lachin JM, Walker EA, Nathan DM (2002). Reduction in the incidence of type 2 diabetes with lifestyle intervention or metformin. N Engl J Med.

[10] Lindström J, Peltonen M, Eriksson JG, Ilanne-Parikka P, Aunola S, Keinänen-Kiukaanniemi S, Uusitupa M, Tuomilehto J (2013). Improved lifestyle and decreased diabetes risk over 13 years: long-term follow-up of the randomised Finnish Diabetes Prevention Study (DPS). Diabetologia.

[11] Gregg EW, Chen H, Wagenknecht LE, Clark JM, Delahanty LM, Bantle J, Pownall HJ, Johnson KC, Safford MM, Kitabchi AE, Pi-Sunyer FX, Wing RR, Bertoni AG (2012). Association of an intensive lifestyle intervention with remission of type 2 diabetes. JAMA.

[12] Lean ME, Leslie WS, Barnes AC, Brosnahan N, Thom G, McCombie L, Kelly T, Irvine K, Peters C, Zhyzhneuskaya S, Hollingsworth KG, Adamson AJ, Sniehotta FF, Mathers JC, McIlvenna Y, Welsh P, McConnachie A, McIntosh A, Sattar N, Taylor R (2024). 5-year follow-up of the randomised Diabetes Remission Clinical Trial (DiRECT) of continued support for weight loss maintenance in the UK: an extension study. Lancet Diabetes Endocrinol.

[13] Clark AM, Hartling L, Vandermeer B, McAlister FA (2005). Meta-analysis: secondary prevention programs for patients with coronary artery disease. Ann Intern Med.

[14] Gudzune KA, Kushner RF (2024). Medications for obesity: a review. JAMA.

[15] Shannon HH, Joseph R, Puro N, Darrell E (2019). Use of technology in the management of obesity: a literature review. Perspect Health Inf Manag.

[16] Cheah KJ, Abdul Manaf Z, Fitri Mat Ludin A, Razalli NH, Mohd Mokhtar N, Md Ali SH (2024). Mobile apps for common noncommunicable disease management: systematic search in app stores and evaluation using the mobile app rating scale. JMIR Mhealth Uhealth.

[17] Peng C, He M, Cutrona SL, Kiefe CI, Liu F, Wang Z (2020). Theme trends and knowledge structure on mobile health apps: bibliometric analysis. JMIR Mhealth Uhealth.

[18] Krebs P, Duncan DT (2015). Health app use among US mobile phone owners: a national survey. JMIR Mhealth Uhealth.

[19] Flores Mateo G, Granado-Font E, Ferré-Grau C, Montaña-Carreras X (2015). Mobile phone apps to promote weight loss and increase physical activity: a systematic review and meta-analysis. J Med Internet Res.

[20] Islam MM, Poly TN, Walther BA, Jack Li YC (2020). Use of mobile phone app interventions to promote weight loss: meta-analysis. JMIR Mhealth Uhealth.

[21] Yen HY, Jin G, Chiu HL (2023). Smartphone app-based interventions targeting physical activity for weight management: a meta-analysis of randomized controlled trials. Int J Nurs Stud.

[22] Ang SM, Chen J, Liew JH, Johal J, Dan YY, Allman-Farinelli M, Lim SL (2021). Efficacy of interventions that incorporate mobile apps in facilitating weight loss and health behavior change in the Asian population: systematic review and meta-analysis. J Med Internet Res.

[23] Chen J, Cade JE, Allman-Farinelli M (2015). The most popular smartphone apps for weight loss: a quality assessment. JMIR Mhealth Uhealth.

[24] Herzig D, Nakas CT, Stalder J, Kosinski C, Laesser C, Dehais J, Jaeggi R, Leichtle AB, Dahlweid F, Stettler C, Bally L (2020). Volumetric food quantification using computer vision on a depth-sensing smartphone: preclinical study. JMIR Mhealth Uhealth.

[25] Page MJ, McKenzie JE, Bossuyt PM, Boutron I, Hoffmann TC, Mulrow CD, Shamseer L, Tetzlaff JM, Akl EA, Brennan SE, Chou R, Glanville J, Grimshaw JM, Hróbjartsson A, Lalu MM, Li T, Loder EW, Mayo-Wilson E, McDonald S, McGuinness LA, Stewart LA, Thomas J, Tricco AC, Welch VA, Whiting P, Moher D (2021). The PRISMA 2020 statement: an updated guideline for reporting systematic reviews. BMJ.

[26] Apiñaniz A, Cobos-Campos R, Sáez de Lafuente-Moríñigo A, Parraza N, Aizpuru F, Pérez I, Goicoechea E, Trápaga N, García L (2019). Effectiveness of randomized controlled trial of a mobile app to promote healthy lifestyle in obese and overweight patients. Fam Pract.

[27] Balk-Møller NC, Poulsen SK, Larsen TM (2017). Effect of a nine-month web- and app-based workplace intervention to promote healthy lifestyle and weight loss for employees in the social welfare and health care sector: a randomized controlled trial. J Med Internet Res.

[28] Carter MC, Burley VJ, Nykjaer C, Cade JE (2013). Adherence to a smartphone application for weight loss compared to website and paper diary: pilot randomized controlled trial. J Med Internet Res.

[29] Fang YY, Lee JI, Wu NY, Chang CI, Huang MC, Lee CY, Huang JY, Lee GGC, Chen CS (2023). Effect of a novel telehealth device for dietary cognitive behavioral intervention in overweight or obesity care. Sci Rep.

[30] Gemesi K, Winkler S, Schmidt-Tesch S, Schederecker F, Hauner H, Holzapfel C (2024). Efficacy of an app-based multimodal lifestyle intervention on body weight in persons with obesity: results from a randomized controlled trial. Int J Obes (Lond).

[31] Laing BY, Mangione CM, Tseng CH, Leng M, Vaisberg E, Mahida M, Bholat M, Glazier E, Morisky DE, Bell DS (2014). Effectiveness of a smartphone application for weight loss compared with usual care in overweight primary care patients: a randomized, controlled trial. Ann Intern Med.

[32] Peksever D, Seçkiner S, Meseri R (2024). Nutrition education via a mobile application on weight loss and quality of life: a randomized controlled trial. Clin Exp Health Sci.

[33] Roth L, Ordnung M, Forkmann K, Mehl N, Horstmann A (2023). A randomized-controlled trial to evaluate the app-based multimodal weight loss program zanadio for patients with obesity. Obesity (Silver Spring).

[34] Jin T, Kang G, Song S, Lee H, Chen Y, Kim S, Shin M, Park YH, Lee JE (2023). The effects of dietary self-monitoring intervention on anthropometric and metabolic changes via a mobile application or paper-based diary: a randomized trial. Nutr Res Pract.

[35] Thorgeirsson T, Torfadottir JE, Egilsson E, Oddsson S, Gunnarsdottir T, Aspelund T, Olafsdottir AS, Valdimarsdottir UA, Kawachi I, Adami H, Bjarnason RG (2022). Randomized trial for weight loss using a digital therapeutic application. J Diabetes Sci Technol.

[36] Turner-McGrievy GM, Wilcox S, Boutté A, Hutto BE, Singletary C, Muth ER, Hoover AW (2017). The Dietary Intervention to Enhance Tracking with Mobile Devices (DIET Mobile) study: a 6-month randomized weight loss trial. Obesity (Silver Spring).

[37] Higgins JPT, Altman DG, Gøtzsche PC, Jüni P, Moher D, Oxman AD, Savovic J, Schulz KF, Weeks L, Sterne JAC (2011). The Cochrane Collaboration's tool for assessing risk of bias in randomised trials. BMJ.

[38] Borenstein M, Higgins JPT, Hedges LV, Rothstein HR (2017). Basics of meta-analysis: I(2) is not an absolute measure of heterogeneity. Res Synth Methods.

[39] Amitai I, Gafter-Gvili A, Shargian-Alon L, Raanani P, Gurion R (2021). Obinutuzumab-related adverse events: a systematic review and meta-analysis. Hematol Oncol.

[40] Khurana T, Klepper C, Fei L, Sun Q, Bramlage K, Arce-Clachar AC, Xanthakos S, Mouzaki M (2022). Clinically meaningful body mass index change impacts pediatric nonalcoholic fatty liver disease. J Pediatr.

[41] Verweij LM, Terwee CB, Proper KI, Hulshof CT, van Mechelen W (2012). Measurement error of waist circumference: gaps in knowledge. Public Health Nutr.

[42] Chew HSJ, Koh WL, Ng JSHY, Tan KK (2022). Sustainability of weight loss through smartphone apps: systematic review and meta-analysis on anthropometric, metabolic, and dietary outcomes. J Med Internet Res.

[43] Antoun J, Itani H, Alarab N, Elsehmawy A (2022). The effectiveness of combining nonmobile interventions with the use of smartphone apps with various features for weight loss: systematic review and meta-analysis. JMIR Mhealth Uhealth.

[44] Unick JL, Beavers D, Jakicic JM, Kitabchi AE, Knowler WC, Wadden TA, Wing RR (2011). Effectiveness of lifestyle interventions for individuals with severe obesity and type 2 diabetes: results from the Look AHEAD trial. Diabetes Care.

[45] Al Naabi Y, Ibrahim N, Dhillon JS (2024). Designing sustainable mobile weight management applications: information technology (IT) experts perspectives. Mhealth.

[46] Motivating patients to use smartphone health apps. Fierce Healthcare.

[47] Bergevi J, Andermo S, Woldamanuel Y, Johansson U, Hagströmer M, Rossen J (2022). User perceptions of eHealth and mHealth services promoting physical activity and healthy diets: systematic review. JMIR Hum Factors.

[48] Pagoto S, Schneider K, Jojic M, DeBiasse M, Mann D (2013). Evidence-based strategies in weight-loss mobile apps. Am J Prev Med.

[49] Wang C, Qi H (2021). Influencing factors of acceptance and use behavior of mobile health application users: systematic review. Healthcare (Basel).

[50] Huntriss R, Salimgaraev R, Nikogosov D, Powell J, Varady KA (2024). The effectiveness of mobile app usage in facilitating weight loss: an observational study. Obes Sci Pract.

[51] Whitson JR (2013). Gaming the quantified self. Surveill Soc.

[52] Nishi SK, Kavanagh ME, Ramboanga K, Ayoub-Charette S, Modol S, Dias GM, Kendall CW, Sievenpiper JL, Chiavaroli L (2024). Effect of digital health applications with or without gamification on physical activity and cardiometabolic risk factors: a systematic review and meta-analysis of randomized controlled trials. EClinicalMedicine.

[53] Dao MC, Thiron S, Messer E, Sergeant C, Sévigné A, Huart C, Rossi M, Silverman I, Sakaida K, Bel Lassen P, Sarrat C, Arciniegas L, Das SK, Gausserès N, Clément K, Roberts SB (2020). Cultural influences on the regulation of energy intake and obesity: a qualitative study comparing food customs and attitudes to eating in adults from France and the United States. Nutrients.

[54] Ghelani DP, Moran LJ, Johnson C, Mousa A, Naderpoor N (2020). Mobile apps for weight management: a review of the latest evidence to inform practice. Front Endocrinol (Lausanne).

[55] Honary M, Bell BT, Clinch S, Wild SE, McNaney R (2019). Understanding the role of healthy eating and fitness mobile apps in the formation of maladaptive eating and exercise behaviors in young people. JMIR Mhealth Uhealth.

[56] Holzmann SL, Holzapfel C (2019). A scientific overview of smartphone applications and electronic devices for weight management in adults. J Pers Med.

[57] Biviji R, Vest JR, Dixon BE, Cullen T, Harle CA (2020). Factors related to user ratings and user downloads of mobile apps for maternal and infant health: cross-sectional study. JMIR Mhealth Uhealth.

[58] Antonelli M, Donelli D (2023). Precision nutrition and artificial intelligence mobile apps: a narrative review. https://iecn2023.sciforum.net/.

[59] Mortazavi BJ, Gutierrez-Osuna R (2021). A review of digital innovations for diet monitoring and precision nutrition. J Diabetes Sci Technol.

[60] Ameryoun A, Sanaeinasab H, Saffari M, Koenig HG (2018). Impact of game-based health promotion programs on body mass index in overweight/obese children and adolescents: a systematic review and meta-analysis of randomized controlled trials. Child Obes.

[61] Patel ML, Wakayama LN, Bennett GG (2021). Self-monitoring via digital health in weight loss interventions: a systematic review among adults with overweight or obesity. Obesity (Silver Spring).

[62] Solbrig L, Jones R, Kavanagh D, May J, Parkin T, Andrade J (2017). People trying to lose weight dislike calorie counting apps and want motivational support to help them achieve their goals. Internet Interv.

[63] Bays HE, Fitch A, Cuda S, Gonsahn-Bollie S, Rickey E, Hablutzel J, Coy R, Censani M (2023). Artificial intelligence and obesity management: an Obesity Medicine Association (OMA) Clinical Practice Statement (CPS) 2023. Obes Pillars.

[64] Ross KM, Wing RR (2016). Impact of newer self-monitoring technology and brief phone-based intervention on weight loss: a randomized pilot study. Obesity (Silver Spring).

[65] Orsama AL, Lähteenmäki J, Harno K, Kulju M, Wintergerst E, Schachner H, Stenger P, Leppänen J, Kaijanranta H, Salaspuro V, Fisher WA (2013). Active assistance technology reduces glycosylated hemoglobin and weight in individuals with type 2 diabetes: results of a theory-based randomized trial. Diabetes Technol Ther.

[66] Fakih El Khoury C, Karavetian M, Halfens RJG, Crutzen R, Khoja L, Schols JMGA (2019). The effects of dietary mobile apps on nutritional outcomes in adults with chronic diseases: a systematic review and meta-analysis. J Acad Nutr Diet.

[67] Schiel R, Kaps A, Bieber G (2012). Electronic health technology for the assessment of physical activity and eating habits in children and adolescents with overweight and obesity IDA. Appetite.

[68] Ernsting C, Dombrowski SU, Oedekoven M, O Sullivan JL, Kanzler M, Kuhlmey A, Gellert P (2017). Using smartphones and health apps to change and manage health behaviors: a population-based survey. J Med Internet Res.

[69] Dent R, McPherson R, Harper ME (2020). Factors affecting weight loss variability in obesity. Metabolism.

