# Supplementary material for: The Role of Mobile Apps in Obesity Management: Systematic Review and Meta-Analysis
Source: J Med Internet Res. 2025 May 6;27:e66887. doi: 10.2196/66887 (PMC12093073; doi:10.2196/66887)
Supplement: Multimedia Appendix 3 [file jmir_v27i1e66887_app3.docx]

| **Study** | **Device name** | **App Components** |
| --- | --- | --- |
| Apinaniz et al (2019) [26] | Smartphone app AKTIDIET® | 1. Goal planning: Users set and track goals for physical activity and nutrition. 2. Self-monitoring: The app allows users to record food intake and exercise sessions to promote awareness and adherence to the program.  3. Behavioral interventions: Includes explanatory videos on the proposed exercises and reinforces the recommendations given during the medical visit.  4. Motivational support: Sends text messages to motivate users, highlighting the benefits of physical activity and the risks of a sedentary lifestyle.  5. Health education: Provides information on the principles of a healthy diet and regular physical activity to improve daily habits. |
| Balk-Møller et al (2017) [27] | Smartphone app SoSu-life | 1. Goal planning: Users choose a specific goal (e.g., weight loss) based on a clinical examination.  2. Self-monitoring: Allows users to record their diet and physical activity, providing personalized feedback and a points system to encourage use of the program.  3. Behavioral interventions: Includes exercise videos to improve physical fitness and structured programs for lifestyle changes.  4. Motivational support: Integrates social elements such as peer challenges and group activities to strengthen mutual support and increase adherence to the program.  5. Health education: Provides practical information on diet, physical activity and general well-being to improve daily habits. |
| Carter et al (2013) [28] | Smartphone app My Meal Mate | 1. Goal Planning: Users can set a weight loss goal and track their daily calorie intake to achieve it.  2. Self-Monitoring: The app allows users to log food intake and physical activities via an electronic food diary, improving awareness and adherence to the program.  3. Behavior Interventions: Behavior reinforcement through personalized weekly text messages based on the user's progress.  4. Motivational Support: Weekly motivational messages to promote goal maintenance.  5. Health Education: Includes a food database and provides information on the principles of a healthy diet and physical activity. |
| Fang et al (2023) [29] | Smartphone app CogniNU | 1. Telehealth Device: The CogniNU app incorporates novel 3D food picture recognition technology combined with cognitive behavioral training programs.  2. Artificial Intelligence: Utilizes AI to analyze food images to identify ingredients and nutrient contents.  3. Cognitive Behavioral Therapy (CBT): Digital program integrating CBT concepts to help users recognize and modify unhealthy eating habits.  4. Nutritional Database: Provides detailed nutritional information and low-fat or low-carbohydrate recipes.  5. Behavioral Monitoring: Tracks eating behavior, mood, physical activity, and sleep quality through self-assessment questionnaires. |
| Gemesi et al (2024) [30] | Smartphone app DiHA “Oviva Direkt für Adipositas” | 1. Self-Management: Participants set daily and weekly goals suggested by the app or created by themselves.  2. Self-Monitoring: Users enter data on nutrition, physical activity, and body weight, receiving automated feedback and reminders.  3. Educational Content: Weekly lessons on obesity, weight loss, and lifestyle recommendations delivered via text, audio, or video formats.  4. User Support: Initial phone call with a coach for app setup and ongoing support through a private chatroom within the app. |
| Laing et al (2014) [31] | Smartphone app MyFitnessPal | 1. Goal planning: Users personalize their goal by choosing the amount of pounds they want to lose per week.  2. Self-monitoring: Food and physical activity logging and calorie counting.  3. Motivational support: The app integrates a social networking function to share progress with friends and reminder notifications to encourage consistency in monitoring.  4. Health education: Provides feedback on food choices, helping users improve awareness of portions, nutrients and eating habits. |
| Peksever et al (2024) [32] | Smartphone app MOtiVE | 1. Nutrition Education: Provides daily messages with dietary advice, motivational tips and practical solutions.  2. Motivational Support: Text, visual, and video messages designed to motivate and educate participants about healthy lifestyle changes. |
| Roth et al (2023) [33] | Smartphone app Zanadio | 1. Multimodal Approach: Combines nutritional guidance, exercise therapy, and behavioral science techniques.  2. Knowledge Transfer: Provides educational content to support users in understanding and managing their weight.  3. Behavior Change: Incorporates strategies to help users modify their behaviors related to diet and physical activity.  4. Motivation and Support: Offers motivational messages and support to encourage adherence to the program.  5. Self-Management Tools: Includes features for tracking weight, diet, and physical activity. |
| Jin et al (2023) [34] | Smartphone app Noom Coach | 1. Dietary Self-Monitoring: Participants used either a mobile application to record their daily food and supplement intake.  2. Nutritional Guidance: The app provided an energy goal to reduce daily calorie intake by 500 kcal. |
| Thorgeirsson et al (2022) [35] | Smartphone app Sidekick | 1. Goal-Setting: Users set and track goals related to nutrition, physical activity, and stress management.  2. Self-Monitoring: The app facilitates tracking of health-related tasks with gamified elements to increase user engagement.  3. Behavioral Interventions: Incorporates elements appetite awareness training that encourages users to eat in response to internal hunger and satiety cues.  4. Motivational Support: Provides instant gratification through rewards and promotes social support through group interactions.  5. Stress Management: Offers relaxation, meditation, and mindfulness exercises to help manage stress and improve decision-making. |
| Turner-McGrievy et al (2017) [36] | Smartphone app FatSecret™ | 1. Goal Planning: Provides a personalized daily calorie goal to support weight loss.  2. Self-Monitoring: Daily food tracking through manual entry, barcode scanning, or food photos.  3. Behavioral Interventions: Includes bi-weekly podcasts based on cognitive behavioral theory to improve program adherence.  4. Health Education: Provides information about nutrition and physical activity to help users develop healthier habits. |
